# Supplementary material for: miR-146a-5p mediates inflammation-induced β cell mitochondrial dysfunction and apoptosis
Source: bioRxiv. 2024 Mar 19:2024.03.18.585543. Preprint. [Version 1] doi: 10.1101/2024.03.18.585543 (PMC10983918; doi:10.1101/2024.03.18.585543)
Supplement: Supplementary file 1 [file NIHPP2024.03.18.585543v1-supplement-4.pdf]

bioRxiv preprint doi: https://doi.org/10.1101/2024.03.18.585543; this version posted March 19, 2024. The copyright holder for this preprint (which was not certified by peer review) is the author/funder. This article is a US Government work. It is not subject to copyright under 17 USC 105 and is also made available for use under a CC0 license.

Supplementary Figure Legends

Figure S1. Pro-inflammatory stress increased miR-146a-5p expression in MIN6 stable cells. (A-C) Fold change values from qRT-PCR experiments (n=6) to determine expression levels of miR-146a-5p in Scr and Scr+Cy MIN6 cells (A), ZIP and ZIP+Cy MIN6 cells (B), and OE and OE+Cy MIN6 cells (C). (D-F) Incucyte live cell image system to track cell death (represented by green or red fluorescence) over time. Cell death was compared between WT and Scr (D), WT and ZIP (E), and WT and OE (F). Scale = 400 µm.

Figure S2. Overexpression of miR-146a-5p increases cleaved caspase-3 levels under pro-inflammatory stress. (A) Western blot image showing abundance of caspase-3 and cleaved caspase-3 proteins in MIN6 cells (n=4) treated with/without pro-inflammatory cytokines. (B) Quantification of western blots. Data is presented as means + SEM and statistical significance is determined by one way ANOVA.

Figure S3. Assessment of mitochondrial architecture in cytokine-treated and untreated MIN6 stable cell lines. (A-F) Comparison of number of mitochondria (A), mitochondria perimeter (B), Feret diameter (C), mitochondria aspect ratio (D), mitochondria circularity (E), and mitochondria roundness (F) across all groups (n=1).

bioRxiv preprint doi: https://doi.org/10.1101/2024.03.18.585543; this version posted March 19, 2024. The copyright holder for this preprint (which was not certified by peer review) is the author/funder. This article is a US Government work. It is not subject to copyright under 17 USC 105 and is also made available for use under a CC0 license.

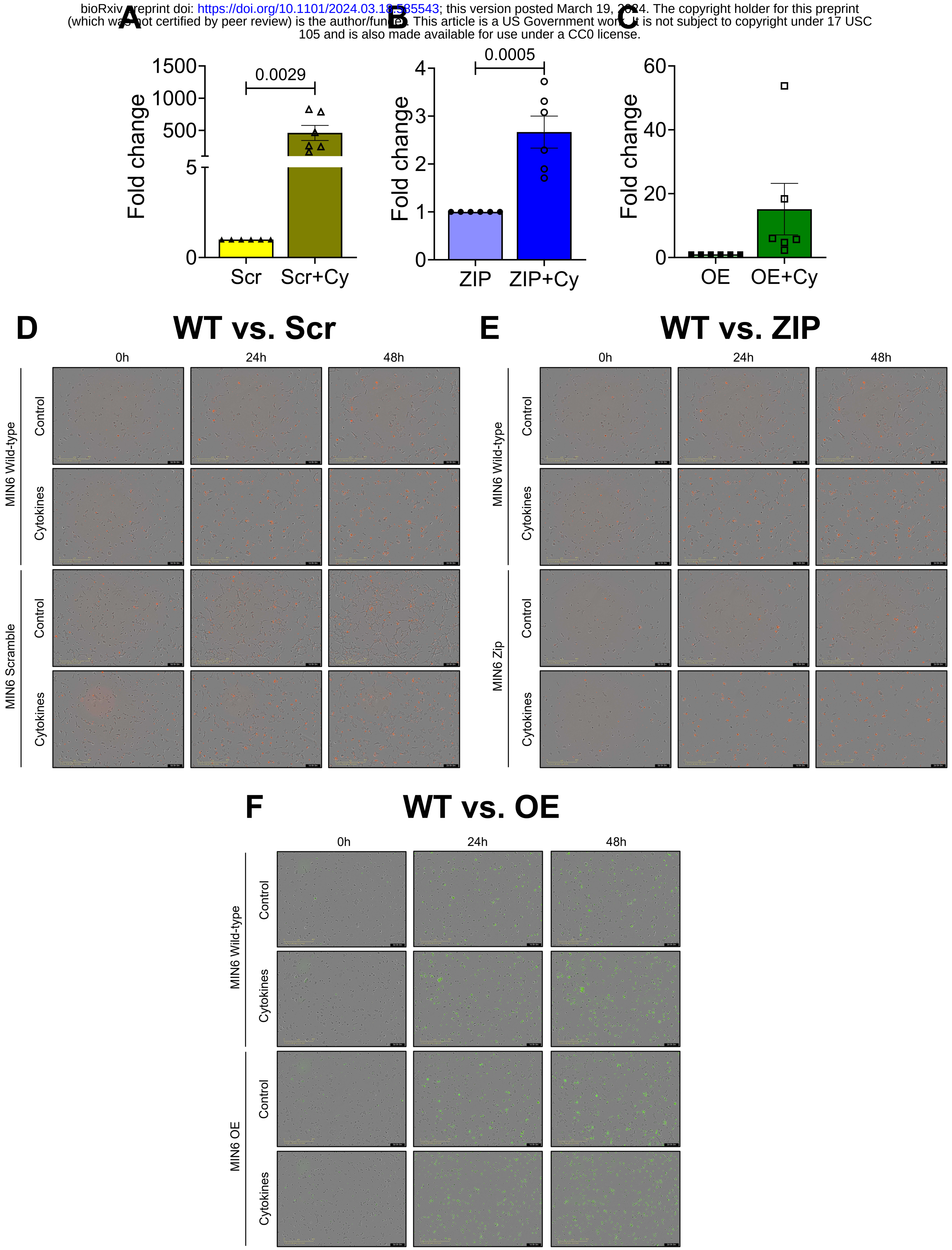

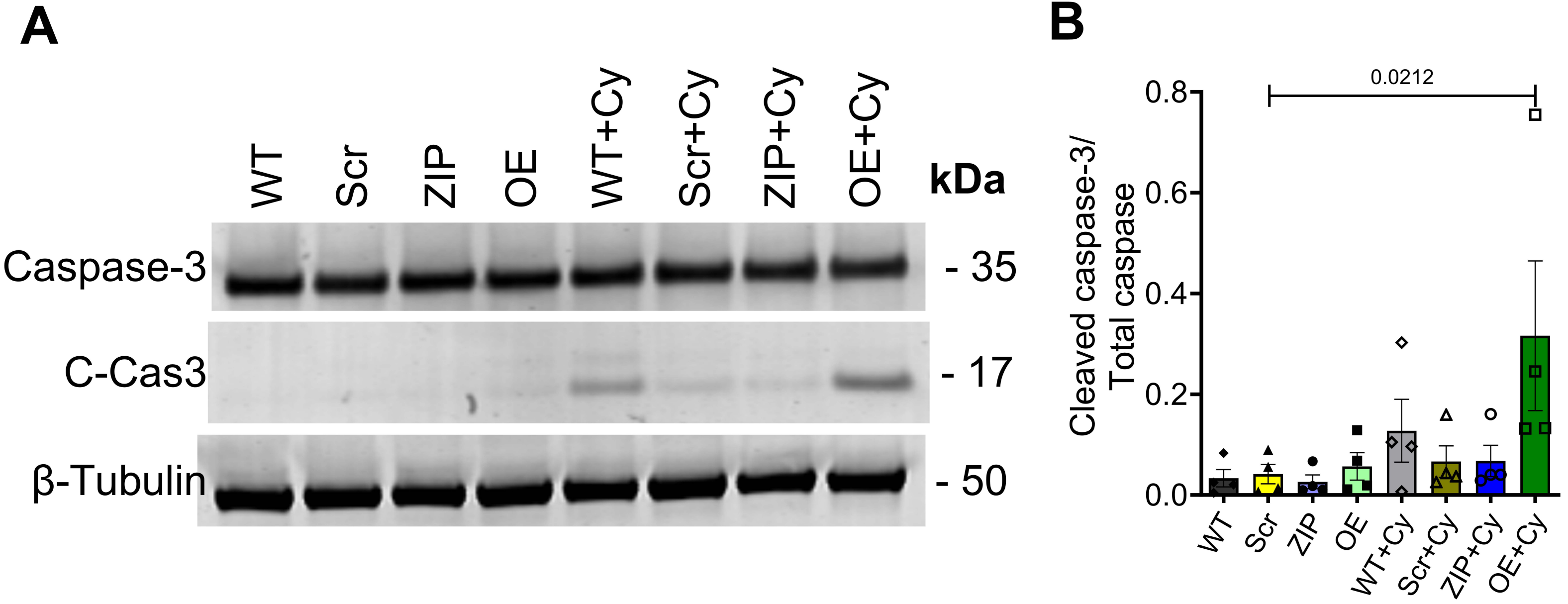
A
WT
Scr
ZIP
OE
WT+Cy
Scr+Cy
ZIP+Cy
OE+Cy
kDa
Caspase-3
- 35
C-Cas3
- 17
β-Tubulin
- 50
B
Cleaved caspase-3/
Total caspase
0.0
0.2
0.4
0.6
0.8
0.0212
WT
Scr
ZIP
OE
WT+Cy
Scr+Cy
ZIP+Cy
OE+Cy

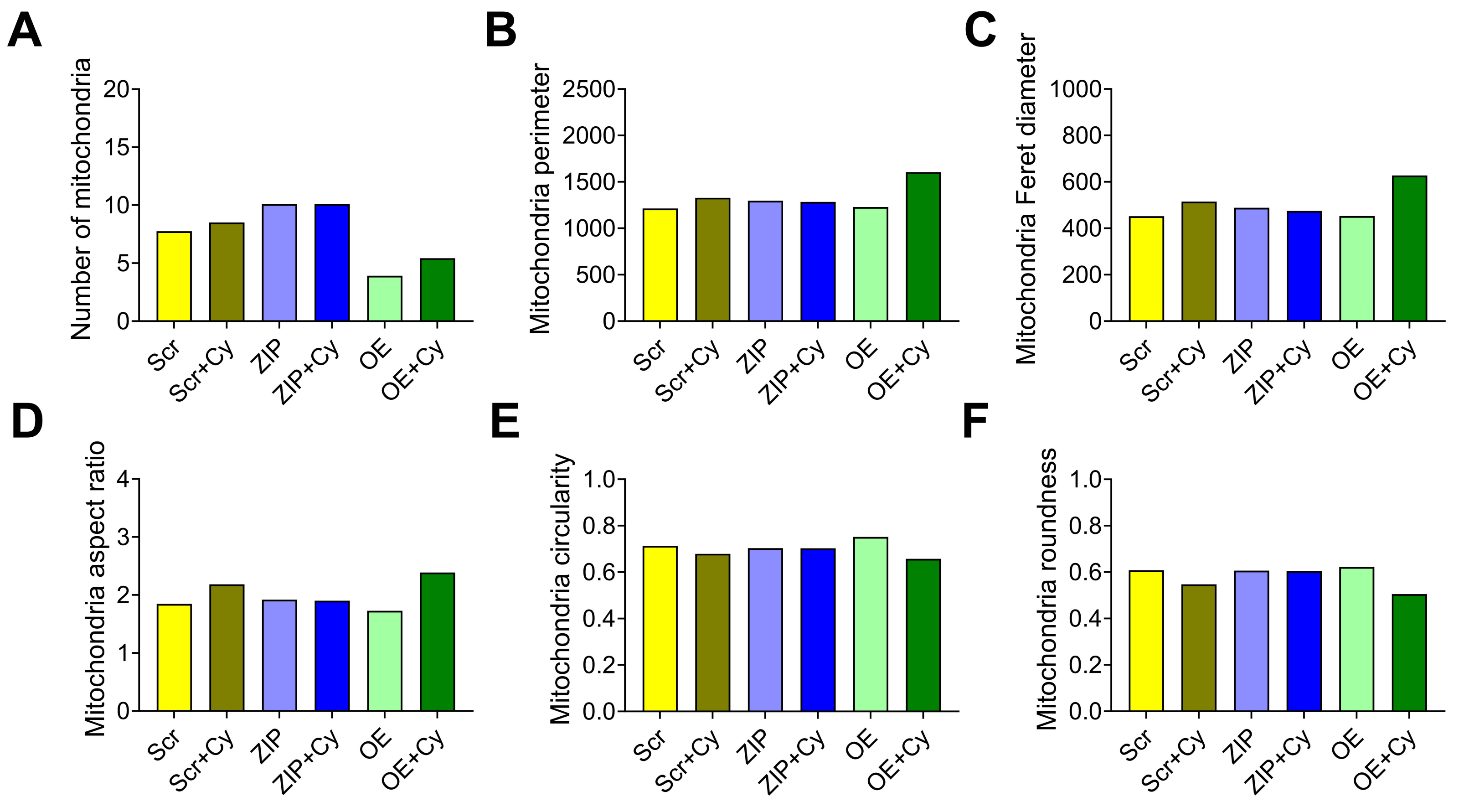
A
B
C
D
E
F
Number of mitochondria
Mitochondria perimeter
Mitochondria Feret diameter
Mitochondria aspect ratio
Mitochondria circularity
Mitochondria roundness
Scr
Scr+Cy
ZIP
ZIP+Cy
OE
OE+Cy
